# Physiology

**Published:** 1860-03

**Authors:** 


### PHYSIOLOGY.

Among the curses of modern times, greater than that of "the yellow-covered literature," or the infidel magazine, or the fiction-crowded monthly, are those innumerable little books, known usually by the heading of this article. They are written by unprincipled men or men so ignorant, that their impudence should be considered a crime. Their object is, by "illustrations," to induce a purchase, and then, by various means, to inflame the imagination and play upon the credulity of the reader first, and his fears next, so that by the time the end of the volume is reached, he is impressed with the profound knowledge of the writer, his undoubted skill, and the necessity of having the benefit of both speedily, and without regard to cost, to remedy existing imagined evils in his own case.

We are in the very frequent receipt of letters from various institutions of learning, showing clearly that the writers have long been laboring under an agony of apprehension as to all sorts of possible ills, and three times out of four with the confession that they have paid their money, sometimes reaching hundreds of dollars without having received the desired benefit. Sometimes, more than that, very often their letters show an amount of mental disquietude to have existed for years, enough to have sent the writers to a lunatic asylum. Some of these letters come from a class of persons which we do not choose to name, and whom we would suppose would be ashamed to be found reading a duodecimo on "Physiology." Parents owe it to themselves to prevent their children from owning such books. In fact, in times like these, when mental poisons are distributed from sources which hitherto were considered respectable, when it is rare to find a publishing-house which is proof enough against the temptation to turn a penny, to refuse to print an article or a book written by a selling name, even if it has a no very hidden squinting towards obscenity or infidelity, we say that in such times, it becomes judicious parents to let both sons and daughters know in plain terms that no book is to be purchased or read by them without its being first submitted to their inspection, and that no newspaper, or magazine, or other periodical should be taken which was not uniformly on the side of the Bible, the Sabbath-day, and a sound morality. But the truth is that there is no magazine published in the United States, in our knowledge, which is suitable for common family reading of a practical, safe, and truthful character, unless they are so decidedly in the interests of a particular religious denomination as to disincline those not of that denomination to patronize it. THE FIRESIDE MONTHLY is such a periodical as one would think would have a wide circulation. It excludes fiction. It contains always plain, practical family articles, striking histories of the actual and true, articles suitable to the young and old, short, pertinent, and pure. It is not in the interest of any religious dénomination, nor does it profess to be a religious publication at all. It is devoted to "science, literature, and practical life," but is by no possibility ever against the Bible, the Sabbath, the ministers of the Gospel, or an evangelical Christianity. It is not only not unacceptable to any sect of Christians, or party of politics, but can not fail to be read with interest and profit by any family, and yet nobody thinks of patronizing such a publication. At the age of six months it has not obtained a hundred subscribers. We make it pay, but no thanks to the public appreciation. We mention this as a striking indication of the taste of the times, of its vitiated character. The rage for pictures and fiction is such that publications which abound in both, sell by scores of thousands every week. These things are not mentioned with any expectation that any special change will take place in consequence of it, but merely as an item of suggestive information to our habitual readers, and with the hope of impressing their minds with the practical fact that as the public taste is so generally vitiated as to the character of its reading, men without any high moral principle will fall in with the current, and will publish whatever will pay, making it necessary for those parents who really love their children to exercise a strict supervision over all they read, and especially the books referred to.

These books first acquaint the boy with the practices referred to, with the almost inevitable result of falling into them, or if they have been learned before, the fears of the reader are so worked upon that the most erroneous impressions are produced, impressions which lead to the injury, literally of "soul, body, and estate." If the mind is disturbed, do not apply to any "Association," to any man at a distance, but to a physician of respectability in your own town, and you will almost always find that your fears are almost if not wholly groundless. Where the trouble does exist, remember this plain fact, no medicine ever cured it. It may suppress for a time, to return inevitably. The only efficient remedy is in the right ordering of the habits of life and in the exercise of force of will.

